# Exploring spirituality and quality of life in individuals who are deaf and have intellectual disabilities

**DOI:** 10.1007/s00127-023-02451-x

**Published:** 2023-03-09

**Authors:** Maria Fellinger, Daniel Holzinger, Jason Fogler, Johannes Fellinger

**Affiliations:** 1 Research Institute for Developmental Medicine, Johannes Kepler University, 4020 Linz, Austria; 2 Institute of Neurology of Senses and Language, Hospital of St. John of God, 4020 Linz, Austria; 3 Institute of Linguistics, University of Graz, 8010 Graz, Austria; 4 Division of Developmental Medicine, Boston Children’s Hospital and Departments of Pediatrics and Psychiatry, Harvard Medical School, Boston, MA 02115 USA; 5 Leadership Education in Neurodevelopmental and Related Disabilities/Institute for Community Inclusion (LEND/ICI), Boston Children’s Hospital, Boston, MA 02115 USA; 6 Division of Social Psychiatry, University Clinic for Psychiatry and Psychotherapy, Medical University of Vienna, 1090 Vienna, Austria

**Keywords:** Spirituality and religion, Quality of life, Deaf, Intellectual disability

## Abstract

**Purpose:**

While positive contributions of religion and spirituality (R/S) to quality of life (QOL) are confirmed by a growing body of evidence, only limited research has involved people with intellectual disabilities and so far, no studies included prelingually deaf individuals with intellectual disabilities. This study explores the role of R/S in people with intellectual disabilities and deafness living in three therapeutic living communities specifically adapted to their needs.

**Methods:**

Forty-one individuals (mean age: 46.93 years, 43.9% female) with prelingual deafness and mild to moderate intellectual disability participated in structured sign language interviews adapted to their cognitive–developmental level, regarding their QOL, individual spirituality and participation in spiritual practices in the community. Participants’ QOL was assessed with an established short measure for QOL (EUROHIS–QOL) adapted to easy-to-understand sign language. With 21 participants, qualitative interviews were conducted. In addition, proxy ratings from caregivers were obtained.

**Results:**

The participants’ ratings of their individual spirituality (*r* = 0.334; *p* = 0.03) and spiritual practices-in-community (*r* = 0.514; *p* = 0.00) correlated positively with their self-reported QOL. Qualitative findings illustrate the importance of R/S and give insights into R/S concepts and practices.

**Conclusions:**

Personal spirituality and participating in spiritual practices are positively related to self-reported quality of life in deaf individuals with intellectual disability (ID). As a consequence, access to spiritual and religious services should be included in comprehensive programs and society at large.

## Introduction

As for any person, individuals with intellectual disabilities (ID) have a right to freedom of thought, conscience and religion, and this freedom is enshrined in various human rights conventions [1]. The notion of spirituality as a basic human need is widely emphasised by scholars, disability advocates, and direct service personnel [2, 3]. The importance of spirituality and religion for holistic care is expressed in the position statement of the World Psychiatric Association [4].

The definition of religion and spirituality is subject to considerable discussion by scholars of various fields [5]. Especially debated are the dichotomy, interwovenness, or indivisibility of spirituality and religion [6–8]. Operationalizations and definitions of either concept include ideas such as relationship with the divine, meaning-making, hope, resilience, and connectedness and contain descriptions of various behaviours and practices [5, 6, 9].

For this exploratory study, we follow Zinnbauer (1999) and colleagues’ [8] assertion that religion and spirituality are overlapping constructs, hereafter referred to as religion/spirituality or R/S. We understand R/S as a search for and relation to the sacred, which must be understood as intertwined with and shaped by beliefs, practices, communities, and institutions. This definition is not only a consensus of different conceptualisations from various religious worldviews; it is also a core of the monotheistic belief system predominant in the research environment. Furthermore, this is backed by several studies discussing R/S mainly in regard to relational components such as attachment and relation to the sacred [9, 10]. A growing body of literature examines the links between R/S, health and well-being, with the majority highlighting the positive effects of R/S on physical health [11–14]. R/S has been found to be positively correlated with mental health and associated with reduced depression, substance abuse, or anxiety [15–19] and to contribute significantly to overall quality of life [20–22]. However, negative aspects of R/S on the health and mental health of people should not be unnoticed [23].

While the mostly positive association between R/S and well-being is established by scholars, the underlying mechanisms and mediating factors remain a conundrum—in part because of challenges in and disagreement about operationalization and overlapping of these concepts [24]. Relationships between these variables are likely to be complex and bi-directional, encompassing cognitive, emotional, behavioural, interpersonal, and physiological dimensions [6]. Factors that have been explored to date include the healthy lifestyle choices supported by religious communities, emotional and material support from the religious community, perceived control, and felt attachment to a benevolent God [9, 25]. R/S can be experienced both as a source of resilience as well as an orienting or motivating factor [26].

Research has thus far rarely addressed the role of R/S in the lives of people with ID, and self-reported perspectives are especially scarce [2, 27]. Studies more commonly focus on the role and use of R/S among the relatives [28] or professional caregivers [27] of people with ID, or the necessity of inclusion of people with ID in religious communities [29, 30]. Studies among people with ID describe predominantly positive effects of R/S, such as higher self-worth, acceptance, and a sense of belonging, and resilience [2, 31]. Furthermore, R/S involvement lessened feelings of stigma and social isolation. R/S practices can especially be of high value in difficult phases of life, such as bereavement [32]. Hence, R/S is found to enhance the QoL [33] in people with ID, especially when linked with inclusion in a religious community [34, 35]. These studies further give valuable insights into the practices, concepts, and understandings of the spiritual lives of people with ID and show their understanding of religious identities, motives, and practices [36–38].

There is limited research on R/S in people with deafness [39] and no research in prelingually deaf people with ID. Due to lack of inclusion in services and such supports as sign language interpretation, and lack of adaptation of R/S teachings to the communicative and cognitive needs of this population, individuals with ID and deafness rarely have access to spiritual and religious counseling. Most individuals with prelingual deafness have experienced insufficient access to language and communication during critical developmental periods in early childhood. This early exclusion from communication is described as *language deprivation syndrome*, which also includes cognitive and socioemotional deprivation with severe impact on these individuals throughout their lifespan [40–44]. Indeed, evidence suggests that the rate of mental health problems in deaf people is at least double the rate of the general population [45].

One often discussed topic for research in the group of people with intellectual disabilities is the issue of effective measurement. Prior studies discuss various barriers in obtaining valid self-reports. Individuals with ID show challenges in understanding questions and giving valid answers due to response biases, impaired theory of mind, or acquiescence [46–49]. Hence, it is a common approach to use proxies in research about people with ID as a means of validation or an additional data source [50, 51]. Mostly parents, caretakers and educators have been the primary informants on the lives of the individuals with ID. Although proxies are able to give accurate information on tangible, objective matters, subjective reports by the individuals themselves are irreplaceable [50, 52]. Addressing people with ID directly in research can have an empowering effect as their views, perspectives and needs are made prominent [53]. The present study, therefore, focuses on self-reports and uses proxies as an additional source of information.

To our knowledge, this study is the first to gather data and analyse the role of R/S in people with ID and deafness. By exploring the role of R/S in the lives of individuals with deafness and ID who are enrolled in therapeutic living community settings specifically adapted to their needs in a cross-sectional study we aim to find relevant perspectives and inspire future research. The aims of this study are as follows:

1. To investigate the role of R/S in the lives of people with ID and deafness.
2. To examine the relationships between R/S and self-reported QoL.
3. To compare self-reports with proxy ratings in regard to R/S and QoL.

## Methods

### Participants and setting

The study was conducted in three specialized therapeutic living communities for deaf people with ID in Austria with a total of 61 individuals (37.7% female; *M*_age_ = 45.67 years, SD = 18.31) with severe-to-profound hearing loss and different degrees of ID. The programs are designed to be fully inclusive, full-time residential and vocational–rehabilitation settings, although 13 participants only make use of the daytime therapeutic workshop programs and live with their families. Length of enrolment in the therapeutic living communities ranged from 6 months to 20 years in our sample.

Of the 61 potential participants, 12 people could not participate in the present study due to their cognitive and communicative limitations. Another eight participants could only be interviewed at one timepoint due to reasons, such as hospitalisation or refusal to participate. Hence, complete data are available for 41 individuals who participated at both timepoints.

The therapeutic living communities place strong focus on the development of social relationships and self-determination through the constant use of individually adapted sign language as well as other forms of visual communication. Accessibility is ensured by mandatory use of sign language by all caregivers as well as through employment of deaf care staff. The development of friendships between staff and participants, as well as friendships with people outside the therapeutic living communities, is welcomed and supported. The work at the therapeutic workshops is important in fostering meaning and sense of purpose, which in consequence positively impacts the development of positive identity and social relationships [54].

Spiritual and religious values are present in the therapeutic living communities, a direct result of the communities’ affiliation to the Catholic hospital of St. John of God and the wider environment’s values and religious systems. Spiritual content is regularly communicated during voluntary morning meetings in which stories from the Christian gospel are told in sign language and real-time-painting conveying prosocial and therapeutic messages about conflict resolution, forgiveness, unconditional acceptance of others, overcoming hardship, and positively contributing to the community, amongst others. It is important to emphasize that the spiritual practices are voluntary for all participants and neither follow a specific denomination, nor entail specific religious liturgy. Respect for religious diversity is deemed essential, and various religious practices (e.g., adherence to specific dietary rules; sharing each other’s holiday traditions) are encouraged [54].

### Measures

#### Intellectual functioning

Intellectual functioning was assessed using two versions of the Snijders–Oomen Non-Verbal Intelligence Scale. As cognitive levels varied, the SON-R 6–40 [55] for individuals with IQ reference age over 6 years equivalent and the SON-R 2½–7 [56] for IQ reference age under this 6-year threshold were used. Given that the SON-R 2½–7 only reports IQ reference age, this parameter is used in this study as a proxy for IQ in this study.

#### Quality of life

To assess QoL, an easy-to-understand Austrian Sign Language version of the EUROHIS–QOL 8-item index was recently developed and used in the study [54]. The short and well-established EUROHIS–QOL was adapted in a multi-step process with input from an interprofessional team. The measure was applied with the deaf population of three therapeutic living communities. In 41 individuals with mild and moderate ID (IQ reference age between 3.3 and 11.8 years) valid self-reported information could be obtained at two timepoints 6 months apart. Test–retest reliability between the two timepoints based on the intraclass correlation coefficient (ICC) was good (ICC 0.83; *n* = 41), and the internal consistency at both timepoints was sufficient (Cronbach Alpha; *n* = 41; *t*1 = 0.81; *t*2 = 0.80).

The study on the adaption of the EUROHIS–QOL-8 also included proxy measures. To optimize our ability to access the participants’ point of view, proxies were asked to answer from the *participants’ perspective* [54].

The self-reported scores on QOL are significantly higher than all three proxy ratings (*p* < 0.05) at both timepoints [54].

#### Assessment of religion and spirituality

The challenge of operationalizing R/S for research becomes magnified in the study population of deaf people with ID. Given the nature of the research topic as well as the specific characteristics of the group a pragmatic mixed-methods approach was employed [57, 58] in which data obtained through a quantitative interview were corroborated and triangulated by results obtained through qualitative interviews with residents. This approach offered the opportunity to gain a nuanced understanding of the role of R/S in the respondents’ lives and how they conceptualized R/S in their own words.

##### R/S quantitative assessment

Our interprofessional team, consisting of a deaf caretaker, a sign language interpreter, a sign competent linguist and a sign-competent neuropsychiatrist, which was the same who adapted the EUROHIS–QOL–ESL for earlier research efforts, considered the use of well-established measures of R/S, such as the WHOQOL–SPRB (WHO QOL–Spirituality, Religiousness and Personal Beliefs) [59] or the Spiritual Well-Being Scale (SWBS) [60]. After an in-depth review and testing of various questions it was decided that due to the specific cognitive and linguistic needs of our sample, R/S should be explored using two questions. In accordance with discussions on the primary dimensions of R/S personal spiritual experience and participation in community demonstrations of spirituality were addressed [21, 61, 62]*.* The two questions were “Do you trust/have faith in God?”^1^ for individual spirituality (inspir), and “Do you like the devotions and praying together?” for community R/S participation (comspir). Those questions are designed to assess the primary dimension of R/S and were adapted based on Plain Language guidelines [63].

Proxy questions in the quantitative survey covered the same two dimensions and asked caregivers to estimate the level of importance that the deaf participants would ascribe to their spiritual life: “To what extent does the individual’s faith help in his/her daily life?”; and “To what extent are the devotions/spiritual morning meetings important to the individual’s life?” Responses were given on a five-point Likert scale.

##### R/S qualitative assessment

The development of the qualitative questionnaire was based on important domains of R/S for people with intellectual disabilities as discussed in the literature: relation to the divine, prayer, [2, 64]; and R/S as a source of support and well-being (SWB, WHO–SPRB) [59]. The questions in sign language have been adapted to the linguistic and cognitive abilities of the population based on Plain Language guidelines and input by the interprofessional research team.

### Procedure and analysis

Quantitative self-reported data (EUROHIS–QOL ESL and the two adapted questions on R/S) were collected at two timepoints (*t*_1_ and *t*_2_) 6 months apart to obtain information on stability of the ratings and test–retest reliability. The qualitative interviews were conducted at *t*_2_.

Data were obtained through standardized face-to-face interviews in which questions could be answered using a 5-point Likert-scale. The interviewers were not directly involved in care, nor were responsible for communicating spiritual content, and did not act as a proxy for participants in this study, but knew the participants well enough to successfully conduct the interviews. Non-involvement in care was considered to be important to curb the effects of possible positive response bias and/or (real or imagined) pressure to acquiesce.

To ensure the validity of the qualitative research, the interviews were transcribed by a native signer not directly involved in conducting interviews. The transcripts were then further analysed thematically by the transcriber and the main author according to Mayring’s [65] method of qualitative content analysis. The coding and systematic categorisation of the data followed a mixed deductive and inductive approach (including themes generated by our team and applicable constructs in the extant literature) and produced the following categories: concepts and understandings of R/S, perceived role of R/S in the participants’ daily lives’, and R/S practices. The translations, meanings and interpretations were further discussed by the above mentioned interprofessional team.

The proxy ratings for R/S were obtained from three different caregivers (proxy 1 and 2 from the therapeutic residential facility, proxy 3 from the day program and workshop facility) at t_1_. Number of proxy ratings differ due to the fact that 12 individuals were only enrolled in the day program.

### Statistical analysis

Pearson correlation was performed to test for correlations between R/S and QoL as well as test–retest reliability between *t*_1_ and *t*_2_. To describe the main variables, univariate analysis was used. The comparison between participants and non-participants was done with a two-tailed *t* test.

## Results

Table 1 provides descriptive statistics for 41 persons completing data collection at both timepoints. Their mean age is 46.93 years (SD 18.07); almost half are female (43.9%). The participants’ mean IQ reference age is 6.87 years (SD 2.08). Forty participants were profoundly deaf. Two individuals (4.9%) had an additional diagnosis of autism, eight persons (19.4%) were diagnosed with epilepsy and eleven persons (26.8%) were diagnosed with cerebral palsy.

**Table 1:** Sample characteristics

Participants, *n*	41
Age in years, M (SD)	46.93 (18.07)
Female %	43.9
IQ reference age in years, M (SD)	6.87 (2.08)
Degree of hearing loss, *n* (%)	
Profound	40 (97.56)
Moderate	1 (2.44)
Autism, *n* (%)	2 (4.88)
Epilepsy, *n* (%)	8 (19.51)
Cerebral palsy, *n* (%)	11 (26.83)
Religious affiliation, *n* (%)	
Christian	37 (90)
Muslim	2 (5)
Non-religious	2 (5)

The 20 non-participants differed significantly (*p* = 0.017) from the participants with regard to their IQ reference age (*M* = 5.39; SD = 1.97), whereas only 16 people were able to complete a cognitive testing.

Table 2 shows the results of the QOL measure and the results regarding R/S for the sample with available self-reports at both timepoints as well as the results of the proxy assessments at *t*_1_.

**Table 2:** Descriptive results for R/S and QOL (means, standard deviations and number of participants included)

	Self-reported R/S *t*_1_	Self-reported R/S *t*_2_	Proxy 1	Proxy 2	Proxy 3
QOL					
M	4.23	4.27	3.88	3.70	3.84
SD	0.65	0.66	0.61	0.75	0.45
*n*	41	41	29	29	39
Individual spirituality (inspir)					
M	4.52	4.37	2.67	2.759	2.93
SD	0.67	0.80	1.06	1.405	1.233
*n*	41	41	29	29	41
Spirituality in community (comspir)					
M	4.19	4.35	2.90	3.138	3.61
SD	1.11	1.01	1.18	1.156	1.070
*n*	40	41	29	29	41

Inspir is individual spirituality, comspir is spirituality in community

Proxy 1 and 2: residential care; Proxy 3: day programme and vocational care

Due to one missing answer in the question on spirituality-in-community the n is smaller (40) in *t*_1_

With respect to reliability and stability of ratings on R/S over time self-reported results regarding spirituality at *t*_1_ correlated positively and significantly with results at *t*_2_ suggesting fair test–retest reliability. The correlation of the ratings for questions on individual spirituality was *r* = 0.388 (*p* = 0.012) and for the question on spirituality in community *r* = 0.543 (*p* = 0.000).

As shown in Table 3, the self-reported information on individual spirituality (Vinspir) correlates significantly with QOL-ratings at *t*_2_ (*r* = 0.334; *p* = 0.03). The self-reported information on spirituality in community (Vcomspir) correlates significantly with self-reported quality of life at *t*_1_ (*r* = 0.438; *p* = 0.01) as well as *t*_2_ (*r* = 0.514; *p* = 0.00). A positive and significant correlation exists between the self-reports and the assessment of proxy 3 regarding the spirituality in community (*r* = 0.36; *p* = 0.02), whereas all other correlations between self-reported R/S and proxy ratings were statistically non-significant.

**Table 3:** Correlations between R/S and QOL ratings

	EUROHIS–QOL–ESL, self-report	QOL Proxy1	QOL Proxy2	QOL Proxy3
*t*_1_				
Vinspir *r*	0.231	− 0.080	− 0.191	− 0.069
* p*	0.15	0.68	0.32	0.67
* n*	41	29	29	41
Vcomspir *r*	**0.438**	0.288	0.327	**0.359**
* p*	**0.01**	0.14	0.09	**0.02**
* n*	**40**	28	28	**40**
*t*_2_				
Vinspir *r*	**0.334**	0.283		
* p*	**0.03**	0.14		
* n*	**41**	29		
Vcomspir *r*	**0.514**	0.345		
* p*	**0.00**	0.07		
* n*	**41**	29		

Pearson correlation coefficient *r*, level of statistical significance *p*, sample size *n*; *t*_1_ timepoint one, *t*_2_ timepoint two; Bolded values are significant

### Qualitative findings

The interviews give valuable insights into the individuals’ understanding of religious concepts and the role of spirituality in their daily lives. In the following section, main findings regarding R/S concepts, role of R/S in the individuals’ daily lives and R/S practices are delineated. Out of the 41 people with quantitative results, 21 qualitative interviews were conducted and analysed. The groups with and without qualitative interview results do not differ significantly in any demographic characteristic (age, gender, IQ reference age) or in results on QOL. As outlined below, “P” refers to participants and “I” to the interviewing investigator.

#### R/S concepts and understandings

The interview transcripts contain spiritual understandings and concepts which overall reflect the input of the spiritual practices in the therapeutic living communities as well as of the local religious community. The expressed notions are in line with Christian religious teaching, such as ideas about heaven or God’s omnipresence:

*P: God is beside the clouds, he can look down on us, but if we look up, we do not see Him. I: Where is Jesus? P: With God.*

*P: Jesus is up there, but you can't see him, he is in heaven*

*P: Jesus is in heaven. […] the teachers at the school [for deaf students] have told me that Jesus is in heaven.*

*P: Jesus? Where? Everywhere? You cannot touch Jesus.*

Some individuals’ notions further contain the specific content of the Gospel stories:

*P: He [God] is with the apostles.*

*P: Jesus preaches, he spreads the message, the apostles follow Jesus.*

*P: Jesus is crucified and in the grave, he is risen, in the past.*

*P: In the evening Jesus [is] resurrected.*

#### R/S in individuals’ daily lives

The interviews provided information about the relevance of spirituality and religion in daily life. For most of the people, a generally positive image of God prevails, who is believed to bless, help, and protect from evil.

*P: He [Jesus] protects, that we stay healthy, Jesus helps and so does God.*

*P: I feel Jesus—Jesus helps, you can feel Jesus, other people in the devotions feel Jesus too. There [in the devotion] many interesting things are told, like about diseases getting healed. You feel the presence of Jesus.*

Beliefs in the divine further seem to guide behaviours or are found helpful in coping with life.

*P: Yes, Jesus is a good friend. […] I: Does Jesus help you? P: Jesus helps, yes, you must not be mean, Jesus likes it when we are good*

*I: Is Jesus your friend? P: Yes, Jesus is allowed to be my friend.*

A resident whose mother died the previous year gave answers pointing to the aspects of R/S`s role in coping with bereavement.

*P: When I am sad, then I pray. My mother and Jesus are above. Yesterday, I prayed that he should come down and should embrace me.*

Some individuals, however, do not attribute an important role to Jesus in their daily lives as they consider Jesus to be dead, absent or invisible.

*I: Does Jesus help? P: I do not know, if Jesus helps, maybe he helps.*

*P: Jesus is crucified, and, in the grave, he is risen, in the past. I: Where is he now? P: I do not know. I: Does Jesus help you? P: No, he died a long time ago.*

Other answers seemed inconsistent.

*I: Is Jesus your friend? P: I do not like Jesus as a friend. I: But Jesus helps you? A: Yes, of course that`s normal.*

*I: Is Jesus your friend? P: I do not know; I wouldn't know if he could sign*

*I: what does Jesus do? P: I do not know. I: No idea? P: No. I: Is Jesus your friend? P: Yes, Jesus is my friend, yes.*

#### R/S practice

Overall, the concept of prayer was understood well. The practice of prayer was generally connected to the spiritual practices in the community taking place in the so called “Andachtsraum” (devotion room). Furthermore, participants report on attendance in services in the local church.

*I: Do you pray? I go to the devotions to pray and chat I would not like to go to the mosque [though].*

*I: Where do you pray? P: I pray at the church there I talk to the priest.*

*P: Yes, I pray when someone dies.*

R/S practices are especially related to certain holidays or phases of life. The interviewees refer to elements and symbols of religious life such as religious holidays like Easter and Christmas, celebrated at the local church.

*I: Do you go to church and pray? P: I sometimes go to church, yes, I go to church at Ash Wednesday, Christmas, and Easter.*

Interestingly some participants address matters of lacking inclusion into the local religious community or dislike of the forms or practices.

*I: Where do you pray? P: downstairs, [in the room for devotions] the problem is that in W. [a local town] they talk, I do not understand when they just talk [in spoken language] I would understand if there was a sign language translator.*

*I: Do you go to our devotions? Yes, I go to the devotions, [but] I want to sleep long – I do not go to the church service in S. [the local town]*

#### Congruency between qualitative and quantitative results

The qualitative questions regarding the friendship with God/Jesus, divine support and the deeds of God/Jesus are a valuable source to estimate the understanding of the quantitative question regarding the individuals’ R/S. The majority (16) were congruent in their positive description of their spiritual lives and the quantitative rating; one person was congruent in the negative assessment. Four individuals gave contradicting answers as they positively rated the importance of R/S but denied relationship to and support from the divine in their lives.

## Discussion

The aim of the study was to explore the role of R/S in a group of individuals with prelingual deafness and ID. To our knowledge this study is the first to analyse the role of R/S within this under-researched population and asserts the notion that people with ID should be addressed directly about their beliefs.

The significant and positive correlations between aspects of individual spirituality and R/S practice in community at the two timepoints indicate that a systematic inquiry was feasible and reliable. An interesting detail in our data is that spiritual practice in community correlates on both timepoints with self-reported QOL but the one on individual spirituality correlates only on timepoint two.

Other than the explanation that the question on spirituality in community is more easily understood as it refers to specific practices, the importance of community for ones’ spiritual life can be one possible reason for this pattern of results. The high positive ratings on spirituality in community show that the R/S activities are a meaningful part of life as they offer opportunities for relationships and room for meaningful rituals. Self and proxy ratings are in better agreement in the ratings on R/S practice in community. One reason for the higher agreement between self and proxy-ratings could be the fact that the devotions are an integral part of the day programme in the therapeutic communities and are joined by the participants and care givers, especially proxies 3 (who, interestingly appeared to be the most aligned with the participants in their ratings, sufficient to produce a statistically significant result). Therefore, the importance ascribed to religious practice, is more often communicated and relatively better known to these caregivers in comparison with personal accounts about the individuals’ beliefs.

The qualitative interviews of our study depict the individuals’ understandings of concepts of R/S as well as the positive role of R/S for the majority of the participants. The responses suggest that R/S is a valuable resource in difficult life circumstances, such as bereavement, gives a sense of protection or guide the individuals’ behaviours. Both the quantitative and qualitative results show that the spiritual care offered for the specific study population is received positively and seemingly contributes to the overall quality of life of the individuals. These positive correlations are in line with results from research in the general population and populations with ID highlighting the positive effects of R/S on mental health, well-being and Quality of Life [13, 19, 20, 52].

### Strengths and limitations

Although response bias and social desirability of answering in self-reports from individuals with ID as discussed in literature [46–49] cannot be excluded, the combination and triangulation of quantitative and qualitative data give valuable insight into the quality and consistency of the assessments. Given the specific cognitive and linguistic profiles of the individuals, both the test–retest as well as the general congruency between quantitative and qualitative data indicate a fair picture of the validity of self-reports. Two-thirds of the qualitative interviews showed a clear congruence with the quantitative ratings regarding the importance of R/S. One possible explanation for the minor incongruences in the qualitative data could be that not all the concepts used were understood fully. As an example, one question contained the metaphor of friendship for the relation to the divine. As discussed in literature, [66] metaphors are processed and understood differently in sign languages than in spoken languages. This is only one example of the difficulty regarding the adequate transfer of abstract concepts into sign language accessible for deaf people with ID and histories of language deprivation in childhood [42].

The linguistic and cognitive characteristics of the study population restricted the choice of instruments and the extent of a possible questionnaire. We are aware that the exploratory questions employed in the present study do not cover the wide range of religious and spiritual experiences, beliefs, and practices. Furthermore, the study was conducted in specific settings catering to the social and communicative needs of people with ID and prelingual deafness and encompassing Christian spirituality. We stress here that this is an exploratory study and make no claims of generalization to the wider population of deaf individuals with ID who may not have access to a supportive living environment, much less one that provides for their R/S needs.

## Conclusion

The high ratings of importance of R/S and the association with QOL in the self-reports of a sample of deaf people with ID indicate the value of accessible spiritual and religious care. Although these findings stem from specialized therapeutic community settings, they suggest that community spirituality is a relevant aspect of quality of life and encourage obtaining self-reports from individuals with special communication needs.

## Footnotes

1 “Do you trust God” is the written and translated version of the question in sign language conveying a broader concept of trust, faith and belief.
